# Chemotherapy-induced niche perturbs hematopoietic reconstitution in B-cell acute lymphoblastic leukemia

**DOI:** 10.1186/s13046-018-0859-3

**Published:** 2018-08-29

**Authors:** Chao Tang, Ming-Hao Li, Ya-Li Chen, Hui-Ying Sun, Sheng-Li Liu, Wei-Wei Zheng, Meng-Yi Zhang, Hui Li, Wei Fu, Wen-Jun Zhang, Ai-Bin Liang, Zhong-Hua Tang, Deng-Li Hong, Bin-Bing S. Zhou, Cai-Wen Duan

**Affiliations:** 1 Key Laboratory of Pediatric Hematology and Oncology Ministry of Health and Pediatric Translational Medicine Institute, Shanghai Children’s Medical Center, Shanghai Collaborative Innovation Center for Translational Medicine and Department of Pharmacology and Chemical Biology, Shanghai Jiao Tong University School of medicine (SJTU-SM), Shanghai, 200025 China; 2 Department of Hematology, Tongji Hospital, Tongji University School of Medicine, Shanghai, 200092 People’s Republic of China; 3 Key Laboratory of Cell Differentiation and Apoptosis of National Ministry of Education, Department of Pathophysiology, SJTU-SM, Shanghai, 200025 China

**Keywords:** Acute lymphoblastic leukemia; chemotherapy-induced niche, Hematopoietic reconstitution, Hematopoietic stem cells, Mesenchymal stem cells

## Abstract

**Background:**

Considerable efforts have been devoted toward the uncovering of the molecular mechanisms underlying the maintenance of hematopoietic stem cells (HSCs) by the normal bone marrow (BM) niche. Previously, we demonstrated that a chemotherapy-induced niche, which is mainly composed of mesenchymal stem cells (MSCs), protects the residual B-cell acute lymphoblastic leukemia (B-ALL) cells from the insult of chemotherapeutic drugs. However, the roles of chemotherapy-induced niche on HSCs functions in B-ALL remain unclear.

**Methods:**

We established an oncogenic *N-MYC*-driven B-ALL mouse model, which were subsequently treated with common chemotherapy drug cytarabine (Ara-C) and daunorubicin (DNR). After treatment, the structures of the BM niche were imaged by immunofluorescence staining. Then, the self-renewal and differentiation capability of the MSCs in the BM after Ara-C and DNR treatment were studied by ex vivo culture and gene expression analysis with RNA-seq and qRT-PCR. The effects of chemotherapy-induced niche on the hematopoietic reconstitution of HSCs were determined with series transplantation assay. Furthermore, the cell cycle, ROS level, mitochondrial membrane potential and cell apoptosis of HSCs were detected by flow cytometry.

**Results:**

The MSCs, which is the main component of chemotherapy-induced BM niche, have decreased self-renewal capability and are prone to differentiate into adipocytes and chondrocytes. The results of gene expression analysis with RNA-seq showed that the MSCs have reduced levels of cytokines, including SCF, CXCL12, ANGPT1, VCAM1, and IL7. Furthermore, the chemotherapy-induced niche perturbed the hematopoietic reconstitution of HSCs in our *N*-*MYC*-driven B-ALL mouse model by promoting HSCs to enter cell cycle and increasing intracellular ROS levels and mitochondrial membrane potential of HSCs, which lead to the cell apoptosis of HSCs.

**Conclusions:**

Chemotherapy-induced BM niche perturbs the hematopoietic reconstitution of HSCs by increasing intracellular ROS level and inducing cell apoptosis.

**Electronic supplementary material:**

The online version of this article (10.1186/s13046-018-0859-3) contains supplementary material, which is available to authorized users.

## Background

Hematopoiesis is a continuous process of orchestrated proliferation, self-renewal, and differentiation of hematopoietic stem cells (HSCs) in the bone marrow (BM) and followed by the egress of mature progeny into the circulating blood [1, 2]. HSCs are the only cells in mammals capable of producing all blood cell lineages throughout life [3]. Chemotherapy may induce hematopoietic deficiency, which is potentially fatal and associated with 20% mortality in patients with acute leukemia or high-grade lymphoma [4, 5]. Up to 30–40% of patients treated with several cycles of chemotherapy failed to mobilize HSCs, and this condition often precludes transplantation and compromises the chance of long-term survival [6, 7].

The BM niche is thought to maintain HSCs stemness while regulating HSCs maturation and differentiation [8, 9]. Although the several genetic studies have provided mechanistic insights into the functions of BM niche cells, mainly MSCs, in the regulation of hematopoiesis during homeostasis [10, 11], the effects of BM niche after stress or injury (such as chemotherapy) on HSCs remains unclear. Chemotherapy exhibits acute cytotoxic effects [12, 13]. In particular, patients that have received several rounds of chemotherapy frequently show irreversible chronic bone marrow damage that often leads to impaired hematopoietic reserve and function [6].

B-cell acute lymphoblastic leukemia (B-ALL) is one of the most common pediatric cancers. Although the treatment of pediatric leukemia has achieved great success owing to the improved regimes of chemotherapy and the five-year overall survival rates of B-ALL exceed 85% in children [14], the chance of relapse and impaired hematopoietic reconstitution after chemotherapy remain major challenges. In our previous study, we demonstrated that a chemotherapy-induced niche shielding residual B-ALL cells can be rebuilt by recruiting BM cells via cytokines secreted from B-ALL cells after chemotherapy [15]. However, whether the chemotherapy-induced niche affects the hematopoietic reconstitution of HSCs in B-ALL is still unknown. Here, we found that chemotherapy-induced niche perturbed the hematopoietic reconstitution in our B-ALL mouse model by dysregulating intracellular ROS levels and inducing cell apoptosis of HSCs. These results rovide a mechanistic explanation for how chemotherapy impairs normal hematopoietic homeostasis, which is important for the consideration of BM transplantation in patients with B-ALL after they undergo chemotherapy.

## Methods

### Animals and B-ALL mouse model

Adult C57/B6 mice (8–12 weeks old) were obtained from Shanghai SLAC Laboratory Animal Co., Ltd. CD45.1/2 congenic strains were bred in our facilities. All animal experiments were approved and monitored by the Shanghai Children’s Medical Center Animal Care and Use Committee. An *N-MYC-*driven B-ALL mouse model was constructed according to the methods described in previous studies [16]. In all experiments, mice were euthanized by CO_2_ asphyxiation. No randomization or blinding was used for the allocation of the experimental groups.

### Chemotherapeutic drug treatment

For the assessment of the role of chemotherapy in BM transplantation, mice were injected i.p. with Ara-C (1 g/kg body weight) or DNR (15 mg/kg body weight) up to 2 days before they were euthanized for analysis.

### Immunofluorescence imaging

Whole-mount tissues and the frozen sections of long bones were prepared as follows: femoral or tibial bones were perfusion-fixed and then post-fixed for 30 min in 4% paraformaldehyde (PFA), incubated in 20% sucrose for cryoprotection, and finally embedded in EBM (embedding buffer). For whole-mount staining, bones were shaved on a cryostat until the bone marrow cavity was fully exposed. Bones were carefully harvested from the melting EBM. Frozen sections were prepared according to the nature protocol [17]. Sections were permeated in 0.1% Triton-X100 dissolved in 1× PBS for 30 min. Then, the sections were blocked in 20% normal goat serum for 1 h. Primary antibodies were diluted and incubated with fixed cells overnight at 4 °C. Next day, the sections were washed for three times in PBS and then incubated with the matching secondary antibodies for 1 h at room temperature. DAPI were then added to the stain nucleus for 10 min. After washing in PBS, the sections were mounted with antifade fluorescence mounting medium. Images were acquired on a Leica SP8 confocal microscope.

### Flow cytometry analysis

Fluorochrome-conjugated or biotinylated mAbs specific to mouse CD45 (clone 30-F11), CD45.1 (clone A20), Ter119 (clone Ter-119), c-Kit (clone 2B8), Sca-1 (clone D7), CD41 (clone MWReg30), CD150 (clone TC15-12F12.2), hematopoietic lineage cocktail, and corresponding isotype controls were purchased from eBioscience. CD31 (clone MEC13.3) and CD48 (clone HM48–1) were from purchased from BioLegend. Cells were analyzed on a LSR II Flow Cytometer (Becton Dickinson).

### Cell culture and differentiation

For the clonal sphere formation, cells were plated at clonal density (< 500 cells/cm^2^) or by single cell sorting into ultralow adherent plates as previously described [18]. Cells were kept at 37 °C and with 5% CO_2_ in a water-jacketed incubator and left untouched for 1 week for the prevention of cell aggregation. One-half medium changes were performed weekly. All spheres in each well were counted at day 9, and results were expressed as a percentage of plated cells. For osteogenic, adipogenic, and chondrogenic differentiation, mouse CD45^−^Ter119^−^CD31^−^PDGFR^+^CD51^+^ cells were treated with StemXVivo Osteogenic, Adipogenic, or Chondrogenic mouse specific differentiation media according to the manufacturer’s instructions (R&D Systems). All cultures were maintained with 5% CO_2_ in a water-jacketed incubator at 37 °C. Osteogenic differentiation indicated by mineralization of extracellular matrix and calcium deposits was revealed by Alizarin Red S staining. Cells were fixed with 4% paraformaldehyde (PFA) for 30 min. After rinsing in distilled water, the cells were stained with 40 mM Alizarin Red S (Sigma-Aldrich) solution at pH 4.2, rinsed in distilled water, and finally washed in Tris-buffered saline for 15 min for the removal of nonspecific staining. Adipocytes were identified by the typical production of lipid droplets and Oil Red staining. Chondrocytes were revealed by Alcian Blue staining, which detects the synthesis of glycosaminoglycans. The cells were fixed with 4% PFA for 60 min, embedded in paraffin, and sectioned. The sections were incubated with 0.5% Alcian Blue (Sigma-Aldrich) in distilled water for 15 min. To remove nonspecific staining, sections were rinsed three times with running water (5 min each).

### Hematopoietic transplantation assay

Competitive repopulation assays were performed using the CD45.1/CD45.2 congenic system. Equivalent volumes of bone marrow cells were collected from an adult mouse. LSKs were transplanted into lethally irradiated CD45.1 recipients with 0.2 × 10^5^ competitor CD45.1 cells. The CD45.1/CD45.2 chimaerism of recipients’ blood was analyzed up to 4 months after transplantation.

**Table:** 

	Forward (5′-3′)	Reverse (5′-3′)
Actin	TCTGGCACCACACCTTCTACAAT	TGGGGTGTTGAAGGTCTCAAA
Cfd	TGCATCAACTCAGAGTGTCAATCA	TGCGCAGATTGCAGGTTGT
Gpnmb	CCCCAAGCACAGACTTTTGAG	GCTTTCTGCATCTCCAGCCT
Ogn	ACCATAACGACCTGGAATCTGT	AACGAGTGTCATTAGCCTTGC
Sp7	ATGGCGTCCTCTCTGCTTGA	GAAGGGTGGGTAGTCATTTG
Acan	CACGCTACACCCTGGACTTTG	CCATCTCCTCAGCGAAGCAGT
Col11a2	TGGCACTCCTGGTCCAGAAG	GCCGGGCTTTCCTGCTA

### RNA extraction and quantitative real-time PCR

Briefly, 500 ng of total RNA was used for cDNA synthesis. Random primers (Qiangen, GM) were used, and all the primer pairs were designed to overlap one intron such that the amplification of genomic DNA is prevented. Q-PCR was sequentially conducted by using SYBR Green PCR master Mix (Takara, JP) and a real-time PCR thermocycler (Angelent), and data were analyzed with the supplied ABI software. Q-PCR was conducted with the following primers:

### RNA-sequencing (RNA-seq) analysis

The cells were sorted into RLT buffer (Qiagen RNAeasy Plus Micro kit), and RNA was purified according to the manufacturer’s instructions. Sequencing libraries were prepared from 10 ng to 100 ng total RNA by using the TruSeq RNA Sample Preparation Kit v2 (Illumina). All the samples were sequenced by Illimina plantform Hiseq X Ten, and all the obtained sequences had a 100 bp-long pair end. Raw sequenced reads were mapped to mouse reference genome hg19 by STAR (version 2.3.0). Fragments per kilobase of an exon model per illion (FPKM) mapped fragments for each gene was generated by cufflinks software. The number of reads mapped to each gene was counted by HTSeq. Differential expressed genes were generated by DESeq2 R package. Genes with p of < 0.01 and |log2(fold change)| of > 2 were defined as differential expressed genes between each groups.

**Table:** 

	A	B	C	D	E	F	G	H	I	J	K	L	M	N
1	Pos	Pos	Neg	Neg	Blank	Axl	Cxcl13	Cd30L	Cd30	Cd40	Crg-2	Ccl27	Cxcl16	Ccl11
2														
3	Ccl24	FasL	Cx2cl1	Gcsf	Gm-csf	Ifn-γ	Igfbp3	Igfbp5	Igfbp6	Il1-α	Il1-β	Il2	Il3	Il3Rβ
4														
5	Il4	Il5	Il6	Il9	Il10	Il12 p40/p70	Il12 p70	Il13	Il17α	Cxcl1	LepR	Leptin	Lix	Cd62L
6														
7	Xcl1	Ccl2	Mcp5	Mcsf	Cxcl9	Ccl3	Mip1r	Mip2	Ccl19	Ccl20	Cxcl4	P-Sel	Ccl5	**SCF**
8														
9	**Cxcl12**	Ccl17	Ccl1	Ccl25	Timp1	Tnfα	TnfRI	TnfRII	Tpo	**Vcam1**	Vegfa	Blank	Blank	Pos
10

**Table:** 

	A	B	C	D	E	F	G	H	I	J	K	L
1	Pos	Pos	Neg	Neg	Blank	bFgf	Cd26	Dtk	E-Sel	FcγRIIb	Flt3L	GitR
2												
3	HgfR	Icam1	Igfbp2	Igf1	Igf2	Il15	Il17Rb	**Il7**	Cxcl11	Cxcl15	Ccl22	Mmp2
4												
5	Mmp3	Opn	Opg	Pro-mmp9	Resistin	Shh-N	Cxcl7	Timp2	Trance	Troy	Tslp	VegfR1
6												
7	VegfR2	VegfR3	Vegfd	Blank	Blank	Blank	Blank	Blank	Blank	Blank	Blank	Pos
8												

### Cytokine array

A commercially available cytokine array of 80 cytokine proteins (Mouse Cytokine Antibody Array C1000; RayBiotech) was used to evaluate cytokine production. Briefly, supernatants obtained from the BM of the Ctrl+A2D and B-ALL+A2D mice were tested for cytokine levels following the manufacturer’s instructions. The intensity of each dot was quantified using ImageJ software from BioRad, and the signals were normalized relative to the background. Array map is showen in the following tables:

### Cell cycle experiment for HSCs

Cell cycle analysis for HSCs was performed as described previously [19]. Bone marrow was harvested by flushing the bone with 1 mL of ice-cold PBS. Then, the red blood cells were lysed once for 5 min at room temperature in ACK buffer and then washed once in ice-cold PBS. The cells were subsequently counted with a hemocytometer. For flow cytometry, the red blood cells were lysed three times for 5 min at room temperature in ACK buffer and washed once in ice-cold PBS and counted in a hemocytometer. For Lin^−^Sca1^+^c-Kit^+^CD48^−^CD150^+^ detection, 10^6^ cells were stained with HSCs surface markers 30 min at 4 °C, then fixed in 4% PFA in PBS for 30 min at 4 °C, washed with PBS, permeabilized with 0.1% Triton X-100 in PBS, and finally stained with anti-ki67-FITC antibody (ebioscience) and DAPI (Life Technologies) at 20 mg/mL for 30 min at room temperature. The cells were washed with washing buffer and analyzed with LSRII flow cytometer (Becton Dickinson).

### ROS staining

For the ROS staining of the HSCs, 5 million cells were stained with HSCs cell surface antibodies as described above. The stained cells were then washed and stained with H2-DCFDA (Life Technologies) for 30 min at 37 °C in Ca^2+^ and Mg^2+^ free HBSS + 2% HIBS + 50 μM verapamil (Sigma-Aldrich). After washing, the cells were analyzed by flow cytometry.

### Apoptosis analysis

Apoptosis was measured by staining freshly harvested bone marrow cells (at least 1 million cells) with Lin^−^Sca1^+^c-Kit^+^CD48^−^CD150^+^ for HSCs, followed by Annexin-V-PE and 7-AAD staining (BD Bioscience). After washing, the cells were analyzed by flow cytometry immediately.

### Mitochondrial membrane potential analysis

Total numbers of cells from bone marrow were stained with HSC markers and then resuspended in 1 ml of PBS. Then cells were incubated with 50 nM DiIC1(5) (Invitrogen) at 37 °C for 15–30 min. After washing, the cells were analyzed by flow cytometry immediately.

### Statistical analysis

All data are presented as the mean ± SD of at least three separate experiments. Statistical analysis was performed using one-way ANOVA followed by Dunnett’s multiple comparison tests. Comparisons between two groups were analyzed using the Student’s t-test. A *p* value of less than 0.05 was statistically significant.

## Results

### Chemotherapy treatment reduces the self-renewal property of MSCs

**Fig. 1:**
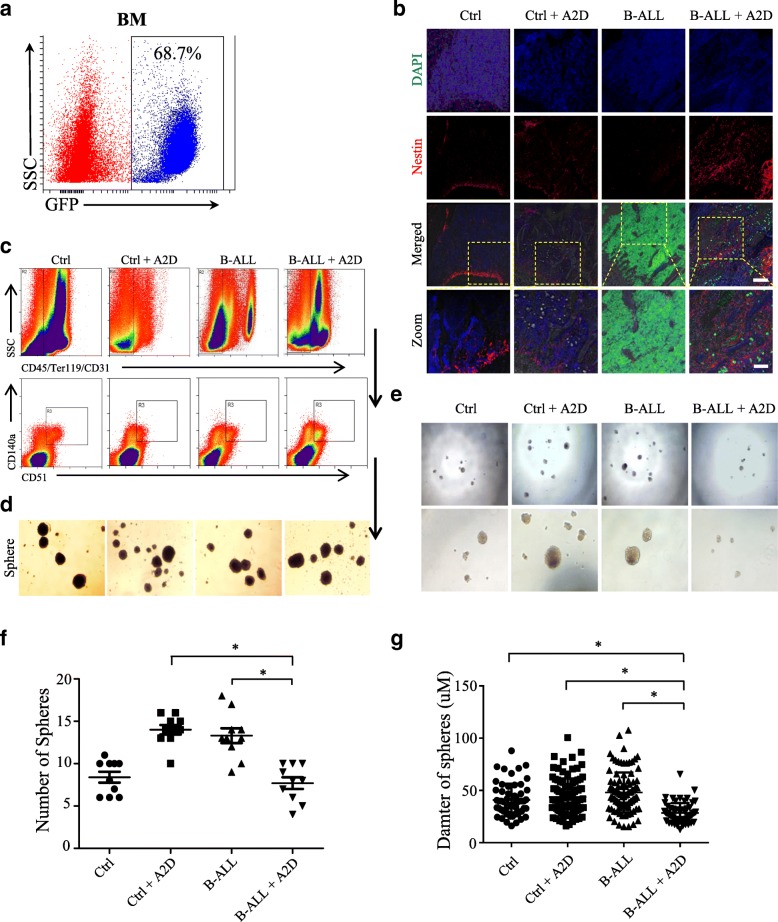
Chemotherapy treatment reduces the self-renewal property of MSCs. **a** Construction of B-ALL mouse model. Representative image of GFP positive cells in *N-MYC-GFP* B-ALL mouse model. **b** Immunofluorescence imaging of nestin in femoral bone marrow (BM) sections of four groups (Ctrl, Ctrl+A2D, B-ALL and B-ALL+A2D). GFP positive cells represent *N-MYC-GFP* derived B-ALL cells, DAPI stains cell nucleus. Scale bars represent 100 μm or 50 μm in inset. **c** Flow cytometric sorting of BM derived MSCs with the markers CD45^−^Ter119^−^CD31^−^CD140a^+^CD51^+^ isolated from the BM of the four groups. SSC, side scatter. **d** Mesensphere-forming assay of the flow-sorted MSCs from the BM of four groups. **e** Representative brightfield images of secondary CD140a^+^CD51^+^ clonal mesenspheres. **f** Statistical summary of the sphere numbers of four groups in (e). **g** Statistical summary of the sphere diameter of four groups in (e). All data in this figure were shown as mean ± SD from three separate experiments. * *P ≤* 0.05; ** *P* < 0.01

To study the morphological structure of BM niche in B-ALL treated with chemotherapeutic drugs, we established the *N-MYC* driven B-ALL mouse model as reported previously [16] (Fig. 1a). Then, we treated the B-ALL mice i.p. with cytarabine (Ara-C) in vivo for two constitutive days (A2D). Consistent with our previous results [15], the cell numbers of Nestin^+^ mesenchymal stem cells (Nes-MSCs) increased dramatically in the B-ALL+A2D group compared with those of the other groups (Ctrl, B-ALL and Ctrl+A2D), as indicated by whole-mount immunofluorescence staining (Fig. 1b). To check whether the properties of the Nes-MSCs were alternated under the stress of chemotherapeutic agents, we sorted the BM-derived MSCs subset out of the total stromal cells (CD45^−^Ter119^−^CD31^−^) from the four groups on the basis of the MSC specific markers CD140a^+^ and CD51^+^ [18] (Fig. 1c). We found that all of the MSCs subset of the four groups is capable of forming replatable mesenspheres in the conditional medium. After dissociation, these MSCs spheres passaged and formed secondary spheres as well, demonstrating the in vitro self-renewal capacity of the MSCs (Fig. 1d). To accurately compare the self-renewal capability of MSCs between these four groups, we dissociated the spheres into single cells and divided the cells into 100 cells per well in 96-wells plates. Interestingly, the capability of sphere-forming of secondary generation showed significant difference between B-ALL+A2D and other groups (Fig. 1e). Therefore, we calculated the numbers and diameter of spheres between four groups and found that the B-ALL+A2D group has dramatically decreased sphere numbers and sphere diameter (Fig. 1f-g), suggesting that chemotherapy treatment remarkably reduces the self-renewal property of MSCs in BM.

### Chemotherapy-induced MSCs are prone to differentiate into adipocytes and chondrocytes

**Fig. 2:**
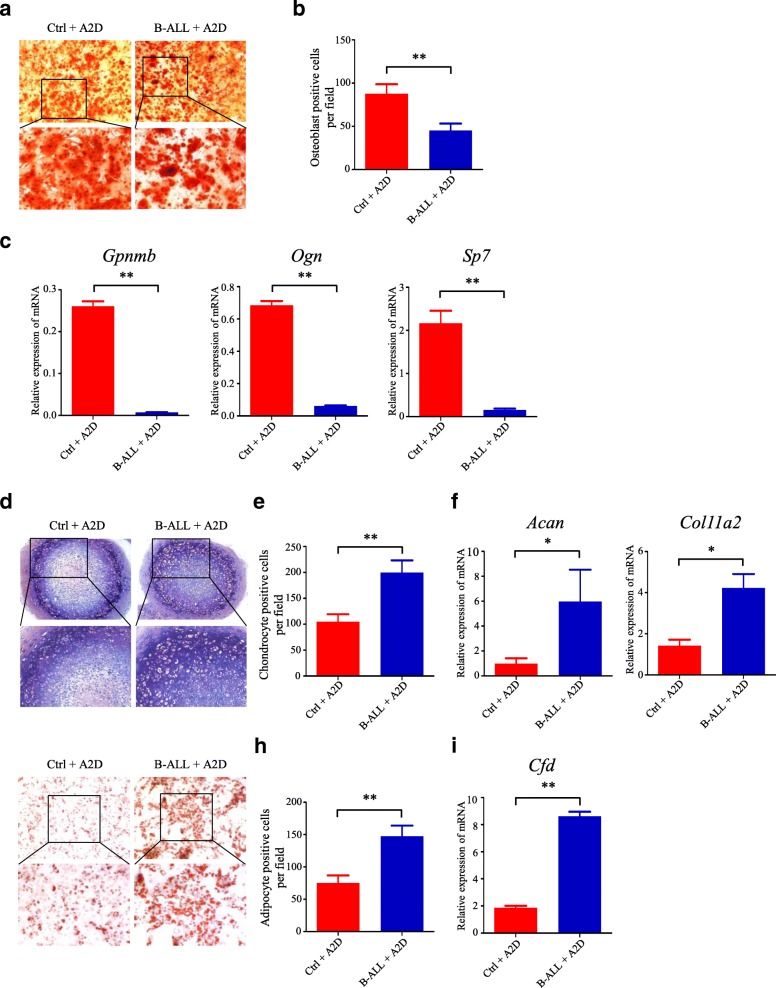
Chemotherapy-induced MSCs are prone to differentiate into adipocytes and chondrocytes. **a** Osteogenic differentiation of MSCs derived from Ctrl+A2D and B-ALL+A2D by Alizarin Red S staining. **b** Statistical summary of osteoblast number per field in (a). **c** The osteoblastic differentiation markers (*Gpnmb*, *Ogn* and *Sp7*) were evaluated by qRT-PCR. **d** Chondrogenic differentiation of MSCs derived from Ctrl+A2D and B-ALL+A2D by Toluidine Blue staining. **e** Statistical summary of chondrogenic cell number per field in (d). **f** The chondrogenic differentiation markers (*Acan* and *Col11a2*) were evaluated by qRT-PCR. **g** Adipogenic differentiation of MSCs derived from Ctrl+A2D and B-ALL+A2D by Oil Red staining. **h** Statistical summary of adipogenic cell number per field in (g). **i** The chondrogenic differentiation markers (*Cfd*) were evaluated by qRT-PCR. All data in this figure were shown as mean ± SD from three separate experiments. * *P ≤* 0.05; ** *P* < 0.01

To further characterize the multilineage differentiation potentials of MSCs under the stress of chemotherapeutic agents, we plated MSCs spheres under in vitro mesenchymal lineage differentiation conditions. Multilineage differentiation was determined by specific staining and morphological and histochemical characterization of mature osteoblastic, chondrocytic, and adipocytic lineage phenotypes after > 30 days of culture. Under conditions optimal for osteoblastic differentiation, clonal mesenspheres from the B-ALL+A2D group differentiated into less alizarin red stain positive osteoblast than that from the Ctr + A2D (Fig. 2a and b). Furthermore, we measured the gene expression levels of osteoblastic differentiation markers by qRT-PCR and found that the expression levels of *Gpnmb*, *Ogn*, and *Sp7* genes were significantly decreased in the MSCs of the B-ALL+A2D group (Fig. 2c). This result demonstrated the impaired potential for osteoblastic differentiation. We also compared the chondrocytic differentiation by culturing MSCs with conditional medium and showed that the clonal mesenspheres of B-ALL+A2D group preferably differentiated into chondrocyte (Fig. 2d and e). The result was subsequently confirmed by the gene expression analysis of the chondrocyte markers *Acan* and *Col11a2* genes (Fig. 2f). We also found that the MSCs of B-ALL+A2D group are prone to differentiate into adipocytes, as demonstrated by oil red staining (Fig. 2g and h) and gene expression analysis of adipocyte markers (Fig. 2i). Overall, these data demonstrate that the chemotherapy-induced MSCs in B-ALL have alternated differentiation potentials and may differentiate into adipocytes and chondrocytes.

### Chemotherapy-induced MSCs have reduced levels of HSC-maintaining cytokines

**Fig. 3:**
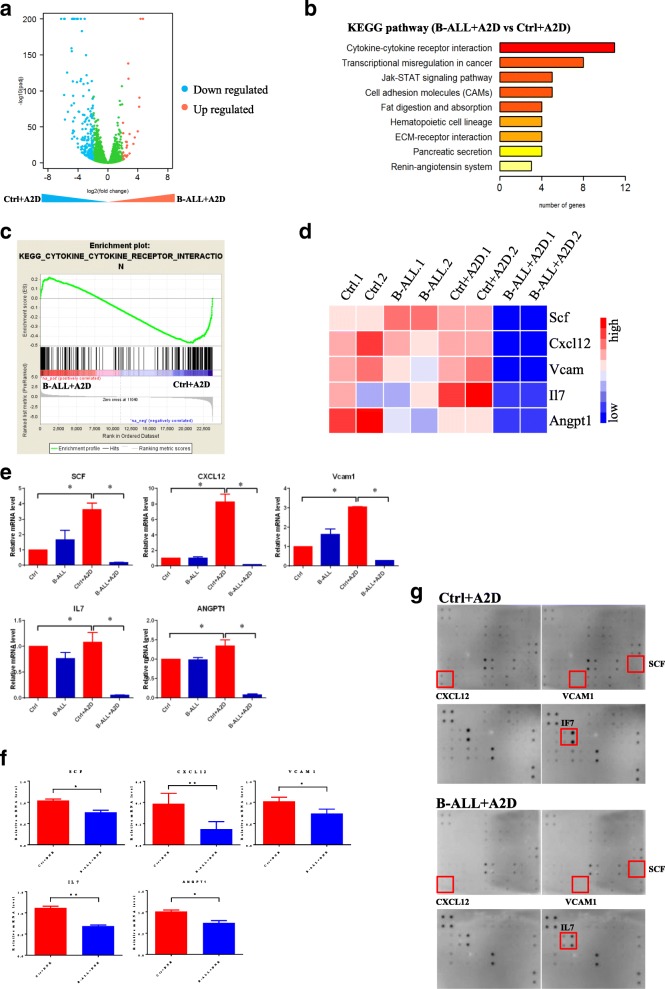
Chemotherapy-induced MSCs have reduced levels of HSC-maintaining cytokines. **a** RNA-seq analysis of differentially expressed genes upregulated and downregulated more than twofold in MSCs of Ctrl+A2D and B-ALL+A2D. Blue dots represent down-regulated genes and red dots represent up-regulated genes. **b** Differentially expressed pathways. B-ALL+A2D versus Ctrl+A2D. **c** Enrichment genes analysis of B-ALL+A2D and Ctrl+A2D. **d** Heat map analysis of SCF, CXCL12, VCAM1, IL7 and ANGPT1 from RNA-seq data. **e** The expression of SCF, CXCL12, VCAM1, IL7 and ANGPT1 in MSCs of Ctrl+A2D and B-ALL+A2D was evaluated by qRT-PCR. **f** The expression levels of SCF, CXCL12, VCAM1, IL7 and ANGPT1 in MSCs of Ctrl+DNR and B-ALL+DNR was evaluated by qRT-PCR. **g** The expression levels of SCF, CXCL12, VCAM1 and IL7 in bone marrow of Ctrl+DNR and B-ALL+DNR was detected by cytokine array. All data in this figure were shown as mean ± SD from three separate experiments. * *P ≤* 0.05; ** *P* < 0.01

To systematically characterize the potential differences between the four groups of chemotherapy-induced MSCs, we performed whole-genome RNA expression profile analysis with the MSCs of Ctrl, B-ALL, Ctrl+A2D, and B-ALL+A2D by total RNA sequencing (RNA-seq). The comparison results among the transcriptome profiles revealed the enrichment of several molecular pathways, such as cytokine-receptor interactions and transcriptional misregulation in cancer, in the B-ALL+A2D group (Fig. 3a-c). As cytokine-receptor interactions are crucial to the maintenance of HSCs through the regulation of the self-renewal and differentiation properties, we analyzed the gene expression of cytokines related to the maintenance of HSCs. Indeed, the MSCs of the B-ALL+A2D group showed significantly decreased gene expression levels of those cytokines, including SCF; cytokines CXCL12, ANGPT-1, and IL7; and vascular cell adhesion molecule-1 (VCAM-1) (Fig. 3d-e). Consistently, those five cytokines were downregulated in the MSCs of B-ALL after DNR treatment (Fig. 3f). As detected by cytokine array, the protein levels of three cytokines (SCF, CXCL12 and IL7) were also decreased in bone marrow of the B-ALL+A2D group compared with those of the Ctrl+A2D group (Fig. 3g). These cytokines regulate HSC maintenance and attraction in the bone marrow [8], suggesting that decreased cytokines expression of MSCs by chemotherapy can disrupt the functions of HSCs and compromise the hematopoietic reconstitution of HSCs.

### Chemotherapy-induced niche perturbs hematopoietic reconstitution of HSCs

**Fig. 4:**
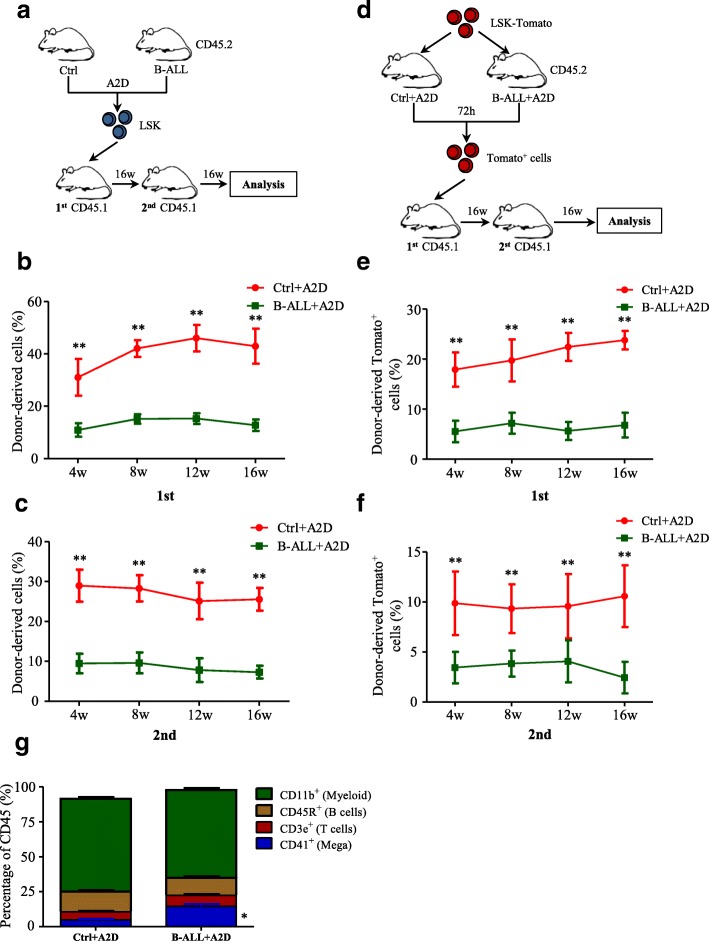
Chemotherapy-induced niche perturbs hematopoietic reconstitution of HSCs. **a** Experimental design to determine the hematopoietic reconstitution of LSK cells isolated from Ctrl+A2D and B-ALL+A2D. **b** Percentages of donor-derived cells after first competitive reconstitution. *n* = 5–6 mice per group. **c** Percentages of donor-derived cells after second transplantation. n = 5 mice per group. **d** Experimental design to determine the hematopoietic reconstitution of normal LSK-tomato^+^ cells isolated from the chemotherapy-induced niche of Ctrl+A2D and B-ALL+A2D. **e** Percentages of tomato^+^ cells after first competitive reconstitution. n = 5 mice per group. **f** Percentages of donor-derived cells after second transplantation. n = 5 mice per group. **g** Flow cytometric analysis of lineage differentiation of LSK in (b). All data in this figure were shown as mean ± SD from three separate experiments. * *P ≤* 0.05; ** *P* < 0.01

Since chemotherapy reduce the expression of HSC-maintaining cytokines in MSCs, we examined the effect of chemotherapy-induced niche on the hematopoietic reconstitution of HSCs with competitive reconstitution assays in two transplantation strategies. First, we sorted the HSCs (Lin^−^Sca-1^+^c-Kit^−^, LSK cells) from the Ctrl+A2D and B-ALL+A2D mice and transplanted them into recipients (CD45.1 mice) with equal numbers of cells (Fig. 4a). The transplantation ratio was analyzed every 4 weeks. The percentage of CD45.2 donor-derived cells from the B-ALL+A2D group was significantly decreased in the first and secondary transplantation (Fig. 4b-c), suggesting the decreased hematopoietic reconstitution capability of HSCs from the B-ALL+A2D group.

To rule out the possibility that the deficiency in the hematopoietic reconstitution of HSCs is caused by the long-term suppression from leukemic cells, we injected normal LSK-tomato^+^ cells into Ctrl+A2D and B-ALL+A2D mice and then sorted the tomato^+^ cells from the chemotherapy-induced BM with flow cytometry in 72 h. The sorted tomato^+^ cells were then transplanted into the recipient mice (CD45.2) (Fig. 4d). The results showed that the percentage of tomato^+^ population from the B-ALL+A2D group decreased dramatically in the first transplantation than those from the Ctrl+A2D (Fig. 4e). Engraftment from the B-ALL+A2D group also showed a remarkable difference in the secondary transplantation (Fig. 4f). Additionally, we analyzed the lineage differentiation of HSCs and found that the HSCs from the B-ALL+A2D group preferentially differentiated into megakaryocytes (Fig. 4g). These results showed that the hematopoietic reconstitution of the HSCs in the B-ALL+A2D group was compromised, at least in part, because of the dramatically decreased HSCs-maintaining cytokines in the chemotherapy-induced niche.

### Chemotherapy-induced niche increases the ROS levels of HSCs and induces cell apoptosis

**Fig. 5:**
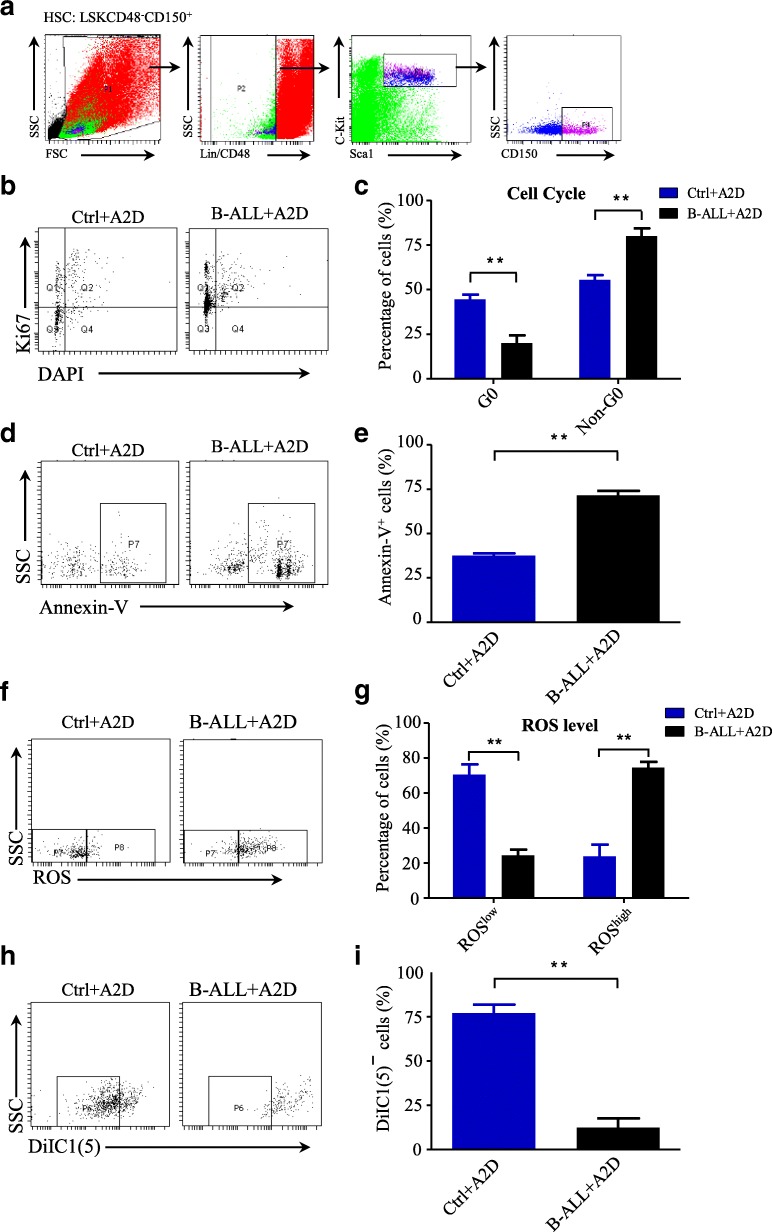
Chemotherapy-induced niche increases the ROS levels of HSCs and induces cell apoptosis. **a** Flow cytometry analysis strategy of HSC. Lineage^−^sca-1^+^c-kit^+^CD48^−^CD150^+^ represent HSCs population. **b** Cell cycle analysis of HSCs in Ctrl+A2D and B-ALL+A2D using Ki-67 and DAPI staining. Representative plots (Lineage^−^sca-1^+^c-kit^+^CD48^−^CD150^+^ gated). **c** Statistical summary of cell cycle distribution in (b). *n* = 3–6 mice per group. **d** The cell apoptosis of HSCs in Ctrl+A2D and B-ALL+A2D was analyzed by flow cytometry. **e** Statistical summary of cell apoptosis ratio of HSCs in (d). n = 3–6 mice per group. **f** The intracellular ROS level of HSCs in Ctrl+A2D and B-ALL+A2D was detected by H2-DCFDA staining. **g** Statistical summary of ROS level distribution in (f). n = 3–6 mice per group. **h** The mitochondrial membrane potential of HSCs in Ctrl+A2D and B-ALL+A2D was detected by DiIC1(5) staining. **i** Statistical summary of mitochondrial membrane potential distribution in (h). *n* = 3–6 mice per group. All data in this figure were shown as mean ± SD from three separate experiments. * *P ≤* 0.05; ** *P* < 0.01

To get the insights of the perturbed hematopoietic reconstitution of HSCs, we studied the HSCs in the Ctrl+A2D and B-ALL+A2D groups with the markers of Lineage^−^sca-1^+^c-kit^+^CD48^−^CD150^+^ as previously reported [20] (Fig. 5a). Firstly, we stained these HSCs with anti-ki67 antibody and DAPI and used flow cytometry to check the cell cycle of the HSCs from the two groups. The flow cytometry results showed that the percentage of HSCs in the G_0_ phase of the B-ALL+A2D group was dramatically decreased than those of the Ctrl+A2D group (Fig. 5b and c). Next, we also compared the cell apoptosis of HSCs in the Ctrl+A2D and B-ALL+A2D groups with staining of annexin V. The results demonstrated that the apoptotic cells in the B-ALL+A2D group drastically increased than those of the Ctrl+A2D group (Fig. 5d and e). Apoptosis was also significantly increased in the HSCs of B-ALL after DNR treatment (Additional file 1: Figure S1a and b). Together, these results suggest that the chemotherapy-induced niche perturbed the hematopoietic reconstitution of HSCs in B-ALL mouse model by promoting HSCs to enter cell cycle and inducing cell apoptosis.

Since oxidative stress is the result of an overabundance of cellular reactive oxygen species (ROS) accumulation formed by the partial reduction of oxygen or a defect in the antioxidant protection mechanism [21]. High intracellular levels of ROS have been showed to limit the lifespan of HSCs and accelerate their differentiation and exhaustion [22]. We speculated that the cell cycle proliferation and increased cell apoptosis of HSCs in the B-ALL+A2D group might be caused by the increased intercellular ROS. To test our inferences, we analyzed the intercellular ROS levels of the HSCs in the Ctrl+A2D and B-ALL+A2D groups using ROS probe. The result showed that, indeed, the HSCs of B-ALL+A2D group had higher levels of intercellular ROS than those of the Ctrl+A2D group (Fig. 5f and g), which is consistent with increased cell proliferation and cell apoptosis in the B-ALL+A2D group. We also found that the ROS level was elevated in the HSCs of B-ALL after DNR treatment (Additional file 1: Figure S1c and d). Furthermore, we measured the mitochondrial membrane potential of the HSCs in the Ctrl+A2D and B-ALL+A2D groups using DilC-5 probe. Consistence with the intercellular ROS levels, the HSCs of B-ALL+A2D group had higher levels of mitochondrial membrane potential than those of the Ctrl+A2D group (Fig. 5h and i). These results indicated that the increasing of intercellular ROS levels and mitochondrial membrane potential at least contributes to the cell cycle and cell apoptosis of HSCs.

Altogether, these results suggest that chemotherapy-induced niche promotes HSCs entering cell cycle and increases cell apoptosis with increased the level of intracellular ROS and mitochondrial membrane potential, which may lead to the reduced hematopoietic reconstitution of HSCs.

## Discussion

Chemotherapy has been shown to damage BM niche and cause myelosuppression, but the underlying mechanism remains controversial [6, 23, 24]. Here, we showed that a chemotherapy-induced niche in B-ALL perturbed the hematopoietic reconstitution of HSCs after transplantation, suggesting an indirect HSC damage mechanism mediated by BM niche. Our results implicated a fundamental role of the chemotherapy-induced niche on HSCs dysfunction and BM failure and demonstrated that chemotherapy agents can alternate the MSCs in the BM. These effects compromised the self-renewal capability of the HSCs. We also suggested that the decrease of cytokines from the MSCs and the increased level intracellular ROS in HSCs contribute to the perturbed hematopoietic reconstitution of HSCs after chemotherapy.

Various cytokines mediate the interaction between HSCs and niche components. Leptin receptor positive perivascular (LepR^+^) cells and Nestin^+^ cells both express and secrete SCF, which is required for the maintenance of HSCs in the bone marrow [10, 11]. CXCL12 derived from mesenchymal progenitors is a niche factor that has been shown to regulate HSC functions, such as retention, quiescence and the ability to induce multilineage reconstitution [25, 26]. Tie2-expressing HSCs situate closely with ANGPT-1-expressing stromal cells, and these interactions have been shown to enhance the adhesion of HSCs to osteoblastic cells through an upregulation of integrinβ1 [27]. Furthermore, angiogenin plays a non-cell-autonomous role in the regulation of hematopoiesis by simultaneously preserving the stemness of hematopoietic stem cells and progenitor cells and promoting the proliferation of lineage-committed myeloid-restricted progenitor cells [28]. Together, these studies highlighted the import roles of different cytokines in BM niche and HSCs maintenance. Here, we showed that these cytokines decreased in the chemotherapy-induced MSCs, and this decrease suggests an alternative myelosuppression mechanism for the maintenance of chemotherapy-compromised HSCs maintenance in a “damaged niche.”

Moreover, HSCs and their niche can be affected by diverse sources of stress, including oxidation, hypoxia, radiation, and chemotherapy, which disrupt HSC homeostasis and regeneration [29, 30]. Our results indicated that chemotherapy-induced niche facilitates the entry of quiescent HSCs into cell cycle and increases cell apoptosis by increasing the level of intracellular ROS and mitochondrial membrane potential, which may lead to the reduced hematopoietic reconstitution of HSCs. These results also suggest a potential indirect mechanism of chemotherapy-induced HSCs failure mediated by MSCs dysfunction. Meanwhile, our results implicated that the chemotherapy-damaged BM niche is not receptive to transplanted HSCs and thus may require the further assistance of HSCs-protective agents [31, 32]. Thus, these HSCs-protective agents may prevent long-term bone marrow failure and could provide a therapeutic option to increase BM function and decrease myelosuppression in chemotherapy-treated patients.

## Conclusion

We report that chemotherapy-induced BM niche perturbs hematopoietic reconstitution through increasing the level of intracellular ROS and inducing the cell apoptosis of HSCs, possibly mediated by reduced cytokines in the niche.

## Additional file

Additional file 1: **Figure S1.** Chemotherapy-induced niche contributes to the increase of ROS and apoptosis in HSCs. (a) HSCs apoptosis in Ctrl+DNR and B-ALL+DNR was analyzed by flow cytometry. (b) Statistical summary of apoptotic cell ratio of HSCs in (a). *n* = 4–8 mice per group. (c) The intracellular ROS level of HSCs in Ctrl+DNR and B-ALL+DNR was detected by H2-DCFDA staining. (d) Statistical summary of ROS level distribution in (c). n = 4–8 mice per group. (PPTX 111 kb)

## Abbreviations

ANGPT-1: Angiopoietin-1
B-ALL: B-cell acute lymphoblastic leukemia
CXCL12: C-X-C motif ligand 12
HSCs: Hematopoietic stem cells
IL7: Interleukin-7
MSCs: Mesenchymal stem cells
ROS: Reactive oxygen species
SCF: Stem cell factor
VCAM-1: Vascular cell adhesion molecule-1
