# Supplementary material for: Chemotherapy-induced niche perturbs hematopoietic reconstitution in B-cell acute lymphoblastic leukemia
Source: J Exp Clin Cancer Res. 2018 Aug 29;37:204. doi: 10.1186/s13046-018-0859-3 (PMC6114852; doi:10.1186/s13046-018-0859-3)
Supplement: Supplementary file 1 — Figure S1. Chemotherapy-induced niche contributes to the increase of ROS and apoptosis in HSCs. (a) HSCs apoptosis in Ctrl+DNR and B-ALL+DNR was analyzed by flow cytometry. (b) Statistical summary of apoptotic cell ratio of HSCs in (a). n = 4–8 mice per group. (c) The intracellular ROS level of HSCs in Ctrl+DNR and B-ALL+DNR was detected by H2-DCFDA staining. (d) Statistical summary of ROS level distribution in (c). n = 4–8 mice per group. (PPTX 111 kb) [file 13046_2018_859_MOESM1_ESM.pptx]

## Slide 1
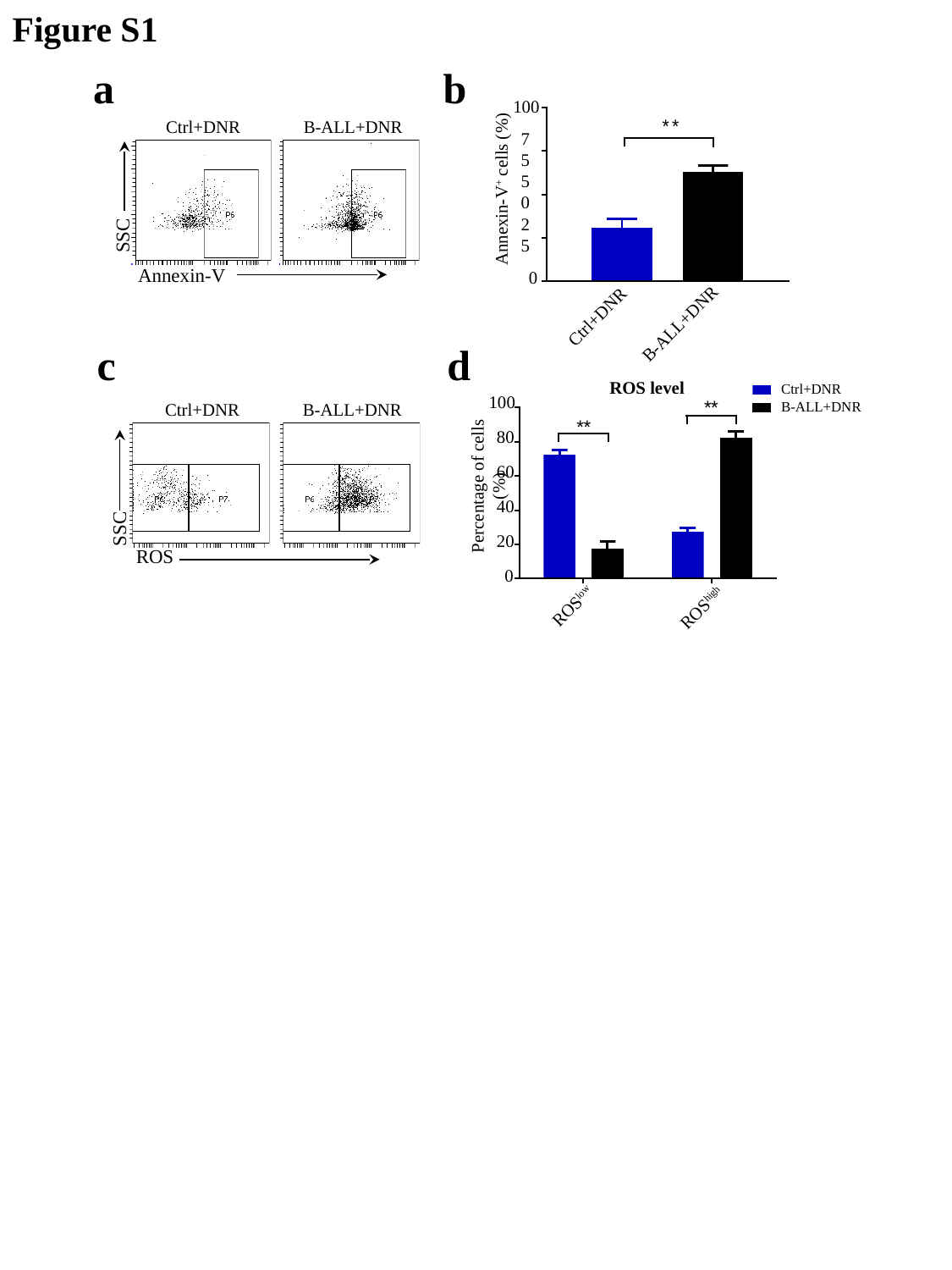

Figure S1
a
b
100
75
50
Annexin-V+ cells (%)
25
0
Ctrl+DNR
B-ALL+DNR
Ctrl+DNR
B-ALL+DNR
SSC
Annexin-V
c
d
ROS level
100
80
60
Percentage of cells (%)
40
20
0
ROSlow
ROShigh
Ctrl+DNR
B-ALL+DNR
SSC
ROS
